# Is ventilation in grocery stores adequate to minimize the risk for airborne transmission of severe acute respiratory syndrome coronavirus 2?

**DOI:** 10.1017/ash.2022.322

**Published:** 2022-11-10

**Authors:** Maria M. Torres-Teran, Jennifer L. Cadnum, Curtis J. Donskey

**Affiliations:** 1 Research Service, Louis Stokes Cleveland Veterans’ Affairs (VA) Medical Center, Cleveland, Ohio; 2 Geriatric Research, Education, and Clinical Center, Louis Stokes Cleveland VA Medical Center, Cleveland, Ohio; 3 Department of Medicine, Case Western Reserve University School of Medicine, Cleveland, Ohio

*To the Editor—*Inadequately ventilated indoor spaces pose a risk for acquisition of severe acute respiratory syndrome coronavirus 2 (SARS-CoV-2) and other respiratory viruses.^1,2^ Therefore, it has been recommended that commercial buildings and schools take measures to assess and improve ventilation.^2^ The Centers for Disease Control and Prevention (CDC) and experts in aerosol science have recommended passive carbon dioxide monitoring as a practical tool to assess ventilation in occupied indoor environments.^2–5^ The concentration of carbon dioxide in outdoor air is ∼400 parts per million (ppm) versus ∼40,000 ppm in exhaled breath.^1^ Thus, carbon dioxide levels rise in occupied spaces that are inadequately ventilated for the number of people present. Carbon dioxide monitoring has been used to assess and improve ventilation in areas such as schools, dental offices, and motor vehicles.^5–7^

Grocery stores provide an essential service and could potentially pose a risk for SARS-CoV-2 transmission.^8^ Based on computational fluid dynamics simulations, the design of ventilation systems in stores may substantially affect the risk of aerosol exposure, with some designs creating local “hot spots” with reduced ventilation and increased risk.^9^ Simulations have also indicated that airflow in grocery stores could enhance dispersal of aerosol particles beyond 2 m of an infected source patient.^8^ Here, we assessed ventilation in several grocery stores in northeastern Ohio using carbon dioxide monitoring.

The study was approved as a quality improvement project by the Cleveland VA Medical Center’s Research and Development Committee. We used carbon dioxide measurements to assess adequacy of ventilation in 10 grocery stores. A member of the research team carried a handheld IAQ-MAX CO2 monitor and data logger (CO2Meter, Ormond Beach, FL) that recorded carbon dioxide levels once per minute during 3–4 shopping trips to each store during busy (defined as lines with 5 or more customers at every checkout counter) and nonbusy (defined as no customers at 1 or more checkout counters) shopping times. The research staff member walked through each of the aisles and shopping areas during each trip, spending at least 3 minutes in each area. Locations in the store and the approximate number of people present were recorded. Carbon dioxide readings >800 ppm were considered an indicator of suboptimal ventilation for the number of people present.^1,2^

Of 10 grocery stores studied, 3 (30%) were classified as large supermarkets (>9,290 m^2^ or >100,000 ft^2^; ≥8 checkout counters), 6 (60%) were classified as medium-sized grocery stores (929–5,017 m^2^ or 10,000–54,000 ft^2^; 3–7 checkout counters), and 1 (10%) was a smaller convenience store that sold groceries (465 m^2^ or 5,000 ft^2^; 1 checkout counter). For shopping trips at nonbusy shopping periods, carbon dioxide levels remained <800 ppm in all 10 stores. During busy shopping periods in the 10 stores, peak carbon dioxide levels increased from 44% to 238% over levels during nonbusy shopping periods, but peak levels only rose to >800 ppm in 2 (20%) stores, both of which were medium-sized supermarkets.

For the 2 stores with carbon dioxide levels >800 ppm, the levels were only elevated in certain areas. Both stores had elevated carbon dioxide levels in the busy checkout areas (peak levels >1,700 ppm). One store also had elevated carbon dioxide levels in crowded aisles. This store had narrow aisles in comparison to the other stores (width 1.2 m vs 3–5 m, respectively) and a lower ceiling height than all other stores except the small convenience store (ceiling height 2.8 m vs 4–8 m, respectively). The figure shows carbon dioxide levels during typical shopping trips to this store during busy and nonbusy shopping periods.

**Fig. 1.**
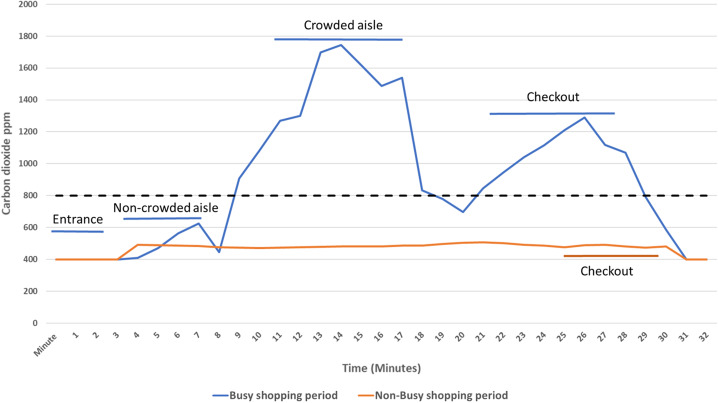
Carbon dioxide levels in parts per million (ppm) in different locations in a grocery store during busy and nonbusy shopping periods. Carbon dioxide levels >800 ppm (dotted lines) were considered an indicator of suboptimal ventilation for the number of occupants present.

Our findings suggest that ventilation in most grocery stores may be adequate to minimize the risk for transmission of airborne pathogens. Our results also demonstrate that carbon dioxide monitoring could potentially be a useful tool to assess ventilation in community settings such as grocery stores. Consistent carbon dioxide levels <800 ppm during busy shopping periods would provide reassurance to customers and employees that ventilation is adequate to minimize risk. If elevated levels are demonstrated during busy shopping periods, interventions could be used to increase ventilation or ensure filtering of recirculated air.^2^ If modifications of the central ventilation system are not feasible, portable air cleaners with high efficiency particulate air (HEPA) filters could be used in areas such as the checkout counter.^10^ Such interventions could reduce the risk to customers and employees working in crowded areas such as checkout counters.

Our study had several limitations. Only 10 grocery stores were studied. We did not record precise numbers of people present during monitoring. We did not determine whether the ventilation systems in the stores included filtering of recirculated air and did not have information on air changes per hour. Filtering can decrease the risk for airborne transmission and is not accounted for by carbon dioxide monitoring.^1^ We did not assess whether airflow patterns in the grocery stores could contribute to dispersal of respiratory droplets beyond 2 meters of an infected source patient. Elevated carbon dioxide levels have not been directly linked to SARS-CoV-2 transmission risk. However, inadequately ventilated indoor spaces are generally considered high-risk areas.^1,2^ Finally, adequate ventilation would not reduce the need for other preventive measures. All stores had signs recommending physical distancing. Masks were optional in all stores but were worn by many customers.
